# NKG2A Expression Is Not *per se* Detrimental for the Anti-Multiple Myeloma Activity of Activated Natural Killer Cells in an *In Vitro* System Mimicking the Tumor Microenvironment

**DOI:** 10.3389/fimmu.2018.01415

**Published:** 2018-06-22

**Authors:** Niken M. Mahaweni, Femke A. I. Ehlers, Subhashis Sarkar, Johanna W. H. Janssen, Marcel G. J. Tilanus, Gerard M. J. Bos, Lotte Wieten

**Affiliations:** ^1^Division of Hematology, Department of Internal Medicine, Maastricht University Medical Center+, Maastricht, Netherlands; ^2^GROW School for Oncology and Developmental Biology, Maastricht University, Maastricht, Netherlands; ^3^Department of Transplantation Immunology, Tissue Typing Laboratory, Maastricht University Medical Center+, Maastricht, Netherlands; ^4^Department of Clinical Genetics, Maastricht University Medical Center+, Maastricht, Netherlands

**Keywords:** natural killer cell, NKG2A, killer immunoglobulin-like receptor, HLA-E, multiple myeloma, tumor microenvironment

## Abstract

Natural killer (NK) cell-based immunotherapy is a promising therapy for cancer patients. Inhibitory killer immunoglobulin-like receptors (KIRs) and NKG2A are required for NK cell licensing, but can also inhibit NK cell effector function. Upon reconstitution in a stem cell transplantation setting or after *ex vivo* NK expansion with IL-2, NKG2A is expressed on a large percentage of NK cells. Since the functional consequences of NKG2A co-expression for activated NK cells are not well known, we compared NKG2A+ vs NKG2A− NK cell subsets in response to K562 cells, multiple myeloma (MM) cell lines and primary MM cells. NK cells were isolated from healthy donors (HLA-C1+C2+Bw4+) and activated overnight with 1,000 U/ml IL-2. NK cell degranulation in subsets expressing KIRs and/or NKG2A was assessed at 21 or 0.6% O_2_. Activated NKG2A+ NK cell subsets degranulated more vigorously than NKG2A− subsets both at 21 and 0.6% O_2_. This was irrespective of the presence of KIR and occurred in response to HLA-deficient K562 cells as well as HLA competent, lowly expressing HLA-E MM cell lines. In response to primary MM cells, no inhibitory effects of NKG2A were observed, and NKG2A blockade did not enhance degranulation of NKG2A+ subsets. KIR− NK cells expressing NKG2A degranulated less than their NKG2A− counterparts in response to MM cells having high levels of peptide-induced membrane HLA-E, suggesting that high surface HLA-E levels are required for NKG2A to inhibit activated NK cells. Addition of daratumumab, an anti-CD38 to trigger antibody-dependent cell-mediated cytotoxicity, improved the anti-MM response for all subsets and degranulation of the KIR−NKG2A− “unlicensed” subset was comparable to KIR+ or NKG2A+ licensed subsets. This demonstrates that with potent activation, all subsets can contribute to tumor clearance. Additionally, subsets expressing KIRs mismatched with the HLA ligands on the target cell had the highest level of activation in response to MM cell lines as well as against primary MM. Our current study demonstrated that if NK cells are sufficiently activated, e.g., *via* cytokine or antibody activation, the (co-)expression of NKG2A receptor may not necessarily be a disadvantage for NK cell-based therapy.

## Introduction

Natural killer (NK) cell-based immunotherapy is an attractive novel therapy against cancer owing to its target selectivity and killing potential (1). NK cells are armed with both activating and inhibitory receptors, and their activation is dependent on the balance between activating and inhibitory signals. The major inhibitory receptors, killer immunoglobulin-like receptors (KIRs) and the NKG2A receptor, provide NK cells with inhibitory signals and are involved in the education process of an NK cell (2, 3). This NK cell education process, also known as licensing, plays a pivotal role in shaping the NK cell ability to kill a target cell. Previous studies on murine NK cells have demonstrated that the number of inhibitory receptors for self-major histocompatibility complex (MHC) expressed on NK cells is proportionate to the strength of NK cell responsiveness against a target cell (4, 5). A more recent study has shown that this is also relevant for human NK cells (6). Moreover, they observed that NKG2A has a stronger licensing impact compared to the KIRs without a significant difference between KIR2DL2/3, KIR2DL1, and KIR3DL1.

In the context of the NK cell response against tumor cells, inhibitory receptors have a dual role: on the one hand, having more inhibitory receptors, and thus better licensed and potentially more potent NK cells, could be advantageous for the NK cell response against MHC/HLA-class I-deficient tumor cells. On the other hand, a licensed NK cell could be inhibited when binding to its cognate ligand expressed on an MHC/HLA-class I competent tumor cell unless an excessive amount of activating signals is present (7). To reduce the inhibitory effects mediated by KIRs, donor-derived, alloreactive, KIR–ligand mismatched NK cells have been proposed as one of the solutions to achieve a better response against tumor cells. Such donor NK cells would namely be fully licensed, albeit, their anti-tumor reactivity would not be hampered due to the genetic incompatibility between donor KIR and patient HLA ligands (8, 9).

In contrast to the KIRs, mismatching for NKG2A and its HLA-E ligand is not possible due to the limited polymorphism of HLA-E. NKG2A can, however, be an important inhibitory receptor for NK cells as it has been shown that NKG2A can inhibit anti-tumor reactivity of NKG2A+ NK cells and NKG2A blocking antibodies could improve the anti-tumor response (10). Moreover, NKG2A is expressed on a large fraction of the NK cells (20–80%) (11, 12), and this percentage is even higher on reconstituting relatively immature NK cells upon allogeneic stem cell transplantation (13). Also, NKG2A has been shown to be overexpressed on NK cells isolated from chronic lymphocytic leukemia patients (14).

Our group aims to develop NK cell-based immunotherapy for multiple myeloma (MM), a hematological malignancy characterized by the growth of malignant plasma cells in the bone marrow for which curative treatment options are currently lacking. We previously reported that both primary MM and MM cell lines express HLA-ABC and HLA-E (15). Additionally, we demonstrated that NKG2A negative KIR–ligand mismatched NK cells were more effective against HLA-class I competent MM cell lines compared to NKG2A negative KIR–ligand matched NK cells (15), also under a more suppressive tumor microenvironment (16). Also, we showed that an antibody-dependent cell-mediated cytotoxicity (ADCC)-triggering antibody, like daratumumab, can enhance the NK anti-MM response and that having a KIR–ligand mismatch can further potentiate the response (16).

Although several of the above-mentioned studies illustrate the functional relevance of either KIRs or NKG2A to set the NK cell activation threshold, the effect of co-expression of these receptors on the single NK cell level remains largely unexplored. As NKG2A is (co-)expressed on many NK cells, including KIR positive subsets, we follow up on our previous findings by investigating whether (co-)expression of NKG2A is beneficial, due to enhanced NK cell licensing, or detrimental due to inhibitory interactions with HLA-E for the NK cell anti-MM response. We compare NK subsets with vs without NKG2A in three different settings: (1) in response to HLA-deficient target cells, (2) in response to HLA competent target cells, and (3) in the presence of ADCC-triggering antibodies. As we intend to perform future clinical studies with activated NK cells and the effect of NKG2A co-expression on activated NK cells remains largely elusive, we activated the NK cells throughout the study with IL-2. Additionally, to explore the influence of tumor microenvironment on the process of NK cell activation, we performed the experiments in the presence of ambient air (21%) or low (0.6%) oxygen concentration. This oxygen concentration is selected from previous experiments (17) and relevant for tumor hypoxia setting.

## Materials and Methods

### Cell Lines and Culture

K562 cell line was cultured in IMDM and 10% fetal calf serum (FCS). UM-9, RPMI8226/s-luc, U266, and RPMI8226/s cell lines were cultured in RPMI1640 and 10% FCS. JJN-3 cell line was cultured in 40% IMDM, 40% low glucose DMEM, and 20% FCS. All cell culture media were supplemented with 100 U/ml penicillin (Gibco) and 100 µg/ml streptomycin (Gibco). K562 and U266 cell lines were purchased from American Type Culture Collection (ATCC, USA). UM-9 and RPMI8226/s-luc cell lines were gifts from Dr. A. Martens, Vrije Universiteit Medisch Centrum, The Netherlands. RPMI8226/s and JJN-3 cell lines were purchased from Deutsche Sammlung von Mikroorganismen und Zellkulturen (DSMZ, Germany). All media were from Gibco, Breda, The Netherlands, and FCS was produced by Greiner Bio-One International, Gmbh. Cell lines were cultured at 37°C in humidified air containing 5% CO_2_ with 21% O_2_ (Sanyo MCO-20AIC, Sanyo Electric Co., Japan).

### Primary MM Cells

Primary MM cells were obtained from the department of cytogenetics as leftover material from a patient subject to a cytogenetic examination. Under the Dutch law on Research Involving Human Subject (http://www.ccmo.nl/en/non-wmo-research), leftover materials from a patient can be used for research and are waived from individual patient’s consent. MM cells were purified using CD138 beads positive selection according to manufacturer’s instruction (Miltenyi Biotech). The purified cells were resuspended in RPMI1640 and 10% FCS supplemented with 100 U/ml penicillin and immediately used in degranulation assay.

### HLA Genotyping, NK Cell Donor Selection, and Analysis of KIR–Ligand Matched/Mismatched Status

The genotypic expression of HLA epitopes relevant for KIR2DL1 (HLA group C2); KIR2DL2/3 (HLA group C1); or KIR3DL1 (HLA-Bw4 and HLA-A*23, -A*24, -A*32) in cell lines and healthy blood donors was determined using Luminex^®^ sequence-specific oligonucleotides analysis (One Lambda). Based on the genotyping result: UM9, U266, and JJN-3 were HLA-C1+C2−Bw4− and RPMI8226/s was HLA-C1+C2+Bw4−. KIR–ligand matched NK cells for UM9, U266, and JJN-3 were, therefore, KIR2DL2/3 positive. KIR–ligand mismatched NK cells for UM9, U266, and JJN-3 were KIR2DL1 positive and/or KIR3DL1 positive. For RPMI8226/s, KIR–ligand matched NK cells were KIR2DL2/3 and/or KIR2DL1 positive. KIR–ligand mismatched NK cells for RPMI8226/s were KIR3DL1 positive. NK cell donors were healthy volunteers or buffy coats with genotype HLA-C1+C2+Bw4+ and phenotypically expressing KIR2DL1, KIR2DL2/3, and KIR3DL1. All donors signed informed consent forms. The use of buffy coats, being a by-product of a required Medical Ethical Review Committee (METC) procedure, does not need ethical approval in the Netherlands under the Dutch Code for Proper Secondary Use of Human Tissue.

### NK Cell Isolation

Natural killer cells were isolated by negative selection of NK cells isolation kit using MACS beads and columns according to manufacturer’s protocol (Miltenyi Biotec, GmbH). For all experiments, NK cells were activated overnight with 1,000 IU/ml recombinant human IL-2 (Proleukin, Novartis) in RPMI-1640 medium (Gibco) supplemented with 10% FCS (Greiner Bio-One), 100 U/ml penicillin (Gibco) and 100 µg/ml streptomycin (Gibco) at 37°C in humidified air containing 5% CO_2_ with 21% O_2_ (Sanyo MCO-20AIC, Sanyo Electric Co., Japan).

### CD107a Degranulation Assay

**Figure 1:**
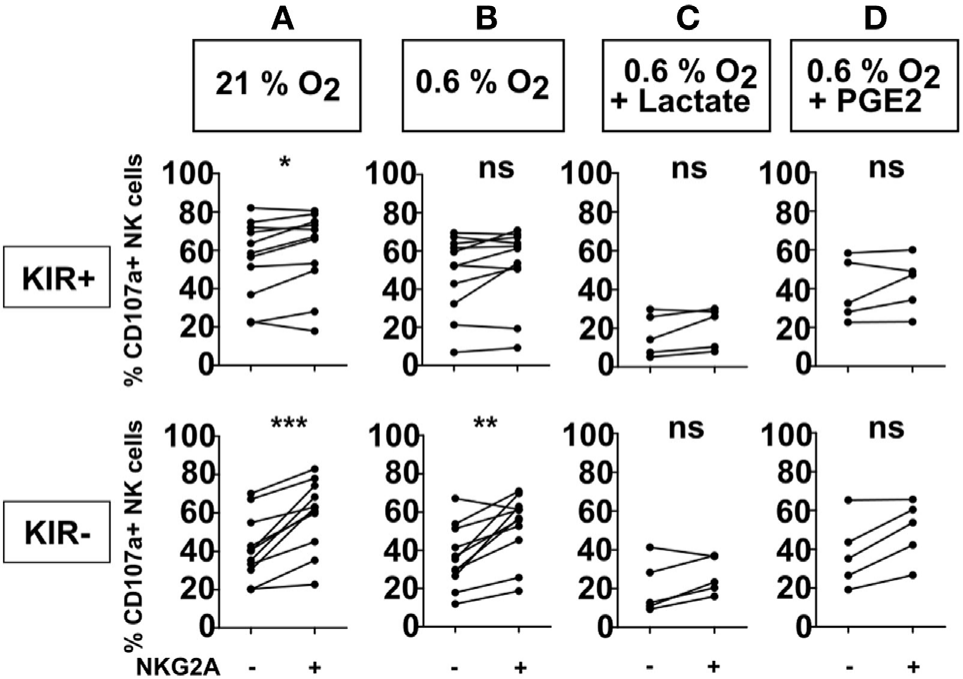
Effect of NKG2A on natural killer (NK) cell degranulation in the presence of different microenvironment factors. NK cells were co-cultured with target cells (K562 cells) in a 1:1 E:T ratio for 4 h at 21% O_2_ **(A)** or 0.6% O_2_ **(B)**, or combinations of 0.6% O_2_ and 50 mM lactate **(C)**, or 100 ng/ml PGE2 **(D)**. Flow cytometry was used to subtype NK cells based on their expression of NKG2A and killer immunoglobulin-like receptors. The percentage of degranulating NK cells is shown as % CD107a+ NK cells. Each dot represents an average of a technical replicate from an individual NK cell donor. **(A,B)** *n* = 11 donors, **(C,D)** *n* = 5 donors tested in independent experiments (ns, not significant, *p < 0.05, **p < 0.01, ***p < 0.001).

To assess NK cell degranulation, CD107a expression on NK cells was analyzed using flow cytometry-based assay. For this, target cells (tumor cells) were plated in 24-well plate at a concentration of 2 × 10^6^ cells/ml per well and incubated overnight at 37°C in humidified air containing 5% CO_2_ with 21% O_2_ (Sanyo MCO-20AIC, Sanyo Electric Co., Japan) or 0.6% O_2_ (except experiment in Figure 5) (Invivo_2_, 1000 Ruskinn Technology Ltd., Bridgend, UK). Prior to the assay, IL-2-activated NK cells were harvested and washed and when indicated in the experiment, subjected to pre-incubation with 50 mM sodium l-lactate (Sigma) or 100 ng/ml prostaglandin E2 (Sigma) or medium (Figure 1; Figure S1 in Supplementary Material), otherwise NK cells were immediately co-cultured with tumor cells in the assay without pre-incubation. For the NKG2A blocking assay, NK cells were incubated with 1 µg/ml anti-NKG2A antibody (clone: Z199, Beckman Coulter) (Figure 3C) or anti-NKG2A-PE-Cy7 (clone: REA110, Miltenyi Biotec) (Figure 5D) for 1 h in 37°C in humidified air containing 5% CO_2_ with 21% O_2_ or 0.6% O_2_ when indicated in the figure. For the HLA-E blocking assay, target cells were incubated with 10 µg/ml anti-HLA-E antibody (clone: 3D12HLA-E, eBioscience) (Figure 5C; Figure S7 in Supplementary Material) for 30 min in 37°C in humidified air containing 5% CO_2_ with 21% O_2_ or 0.6% O_2_ when indicated in the figure. For the ADCC assay (Figure 4), tumor cells were pre-incubated for 30 min with 1 µg/ml daratumumab or medium at 21% O_2_ or 0.6% O_2_ before co-cultured with NK cells. NK cells exposed to tumor microenvironmental factors (TMEF) were then, in duplicate wells, co-cultured with the target cells and 2 µl anti-CD107a-Horizon V450 (clone: H4A3, BD) was added per well. After 1 h of co-culture, monensin (BD) was added. After another 3 h, the plate was placed on ice to stop the reaction. Cells were then stained on ice with anti-CD3-APC/H7 (clone: SK7, BD), anti-CD56-PeCy7 (clone: B159, BD), anti-KIR2DL1-APC (clone: 143211, R&D), anti-KIR2DL2/3/S2-PE (clone: DX27, Miltenyi Biotec), anti-KIR3DL1-FITC (clone: DX9, Miltenyi Biotec), and anti-NKG2A-PC5.5 (clone: Z199, Beckman Coulter). To analyze different subsets of NK cell, CD3−CD56+ cells were gated followed by gating of NKG2A− and NKG2A+ population and further gating based on the KIRs expressions.

### Induction of HLA-E Expression Using HLA Leader Peptides

U266 cells were incubated with 500 µM of HLA-A1 (VMAPRTLLL), HLA-B7 (VMAPRTVLL), or a non HLA-E binding control peptide (RGPGRAFVTI) (Biosynthesis Inc.) overnight at 37°C, 21% O_2_ as previously described (15, 18). Additional negative controls were included by incubating U266 cells in DMSO, the peptide’s solvent or in the medium. After the incubation, HLA-E expression was determined by flow cytometry by staining the cells with an HLA-E antibody (clone: 3D12 HLA-E, eBioscience) or with a matched isotype control, mouse IgG1 kappa (clone: P3.6.2.8.1, eBioscience). Following the induction, U266 cells were used in the CD107a assay (Figure 6) as described in the previous section.

### Flow Cytometry

Cells were washed with PBS (Gibco) and stained first for dead cells using Live/Dead^®^ Fixable Aqua Dead Cell Stain Kit (Molecular Probes™, USA) for 30 min on ice in the dark. Cells were further washed with FACS buffer (PBS, 1% FCS) and stained with antibodies for 30 min on ice in the dark. All flow cytometric analyses were performed with BD FACS Canto II. Data were analyzed with FlowJo 10.1r5 64 bit software.

### Statistical Analysis

All statistical analysis was performed with GraphPad Prism V software (Graphpad Software Inc., San Diego, CA, USA) using non-parametric *t*-test with repeated measure (Wilcoxon signed rank test). * indicates a *p*-value of <0.05 and ** indicates a *p*-value of <0.01, and *** indicates a *p*-value of <0.001.

## Results

### Expression of the Inhibitory NKG2A Receptor Could Be Advantageous for IL-2-Activated NK Cells Against HLA Negative Tumor Cells

To investigate the effect of NKG2A expression on the anti-tumor response of IL-2-activated NK cells against HLA-class I negative target cells, we performed a flow cytometry-based degranulation (CD107a) assay by co-culturing NK cells and HLA-class I negative K562 cells followed by staining for KIRs and NKG2A to enable NK subset analysis. Under normal laboratory conditions of 21% O_2_, a slightly higher percentage of degranulating (CD107a+) NK cells was observed for the subsets expressing NKG2A as compared to NKG2A negative counterparts, and this was observed for both KIR+ (average increase 4.6%) and KIR− (average increase 18%) subsets (*p* < 0.05 and *p* < 0.001, respectively) (Figure 1A). As the tumor microenvironment could potentially impair cytolytic effector function of the NK cells, co-cultures were also performed in the presence of biochemical context mimicking tumor microenvironment, i.e., in the presence of 0.6% O_2_, or in the combination of hypoxia with 50 mM lactate or 100 ng/ml prostaglandin E2 (PGE2). At 0.6% of O_2_, we observed more degranulation in NKG2A-expressing NK cells than for the subsets not expressing KIRs (average increase 16.3%) (Figure 1B). However, for KIR+ subsets there was no difference in the percentage of degranulating NK cells with vs without NKG2A (average increase 4.5%) (Figure 1B). In the conditions where 0.6% O_2_ was combined with PGE2 (average increase 11.8% for KIR− and 3.7 for KIR+) or lactate (average increase 6.1% for KIR− and 4.2% for KIR+), however, this did not reach significance (Figures 1C,D). In the absence of target cells, the percentage of NK cell degranulation was very low (Figure S1 in Supplementary Material). Nonetheless, we also observed a similar pattern as in conditions with target cells with slightly higher percentages of degranulating NKG2A positive NK cell subsets. Of note, in none of the donors, NKG2A expression levels by the NK cells were clearly influenced by 4 h co-culture of NK cells and K562 in the presence of hypoxia, lactate, or PGE2 (Figure S2 in Supplementary Material). Altogether, these results suggest that against an HLA negative tumor cell line, the presence of the NKG2A receptor, especially on KIR− subsets could be beneficial for IL-2-activated NK cells also in the presence of more suppressive microenvironmental factors, presumably because these NK cells were better licensed.

### NKG2A Does Not Inhibit the Response of IL-2-Activated NK Cells Against Myeloma Cell Lines Expressing Low Levels of HLA-E

**Figure 2:**
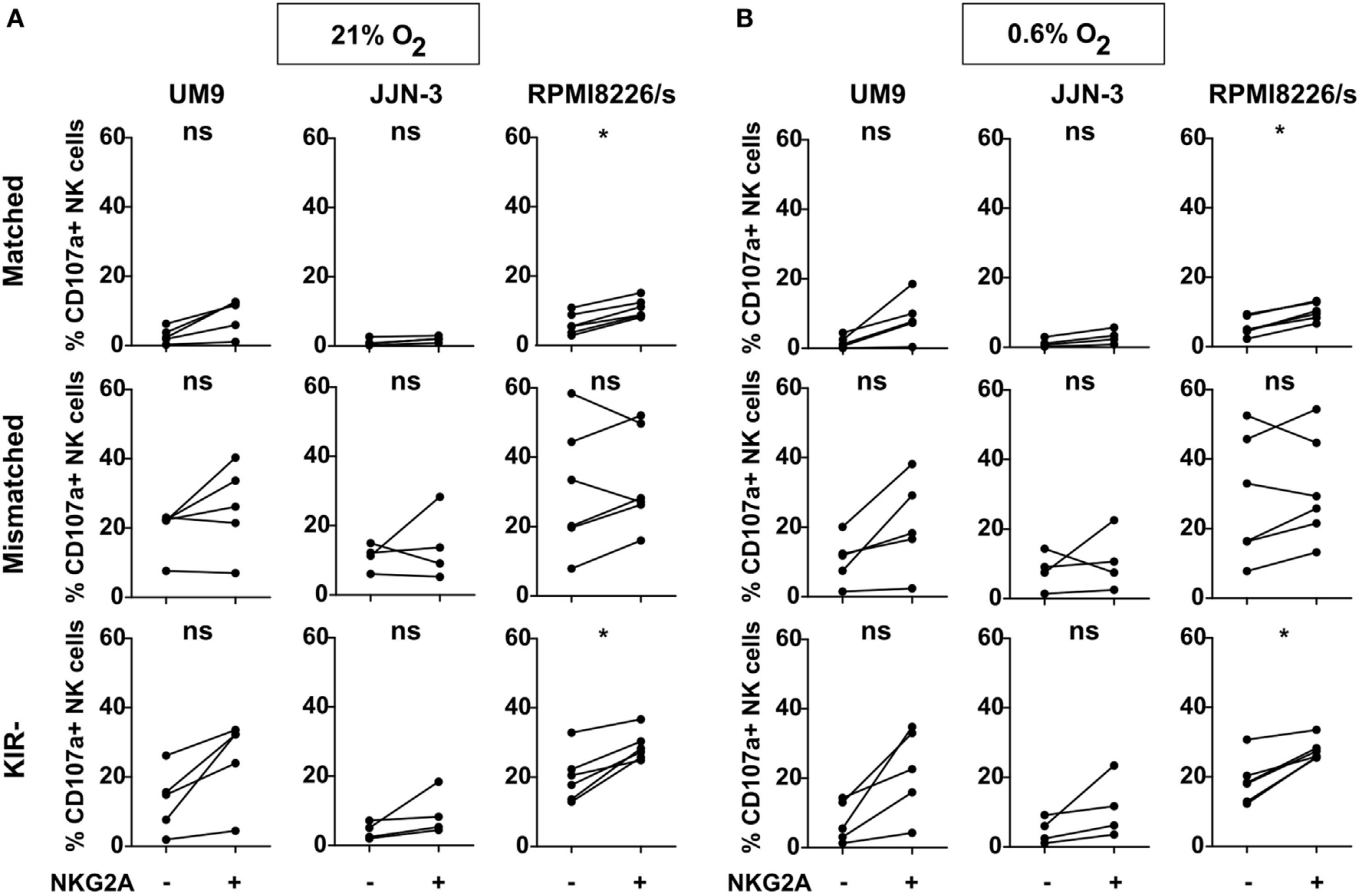
NKG2A and killer immunoglobulin-like receptors (KIR) subset analysis per cell line. Natural killer (NK) cells were co-cultured with UM9, RPMI8226/s, or JJN-3 cells in a 1:1 E:T ratio for 4 h in 21% O_2_ **(A)** or 0.6% O_2_ **(B)**. Flow cytometry was used to subtype NK cells based on their expression of NKG2A and KIRs. Degranulating NK cells were denoted as CD107a+ NK cells. Each dot represents an average of a technical replicate. *n* = 5 independent experiments with five different donors (UM9), four independent experiments with four different donors (JJN-3), and six independent experiments with six different donors (RPMI8226/s) (ns, not significant, *p < 0.05).

The interaction between the NKG2A receptor and its ligand, HLA-E, can have an inhibitory effect on the NK cell anti-tumor capacity and could outweigh the beneficial effect of improved licensing. Therefore, we investigated the effect of NKG2A on IL-2-activated NK cell degranulation in response to three MM cell lines (UM9, RPMI8226/s, and JJN-3) expressing both HLA-class I and HLA-E (Figure S3 in Supplementary Material). Based on the HLA genotypes for classical class I of the cell lines, NK cells were divided into subsets expressing: 1) no KIRs, 2) KIRs that are KIR–ligand matched, or 3) KIRs that are mismatched for the HLA ligands on the target cells. We subsequently compared the response of NK cells (co-)expressing NKG2A vs NK cells lacking NKG2A for each of the three groups. In the absence of target cells, the percentage of degranulating NK cells was negligible (Figure S4 in Supplementary Material). We observed that for the matched and the KIR negative subsets, the percentage of degranulating NKG2A positive cells was slightly higher than degranulation of the subsets lacking NKG2A in most donors. This difference reached significance when NK cells were co-cultured with RPMI8226/s both in the presence of 21% O_2_ or 0.6% O_2_ (Figure 2). For NK cells expressing mismatched KIRs, we did not observe a difference between NKG2A+ vs NKG2A− cells against all cell lines. Although the three cell lines tested in this study expressed HLA-E, albeit at low levels (Figure S3 in Supplementary Material), in only 4 out of 45 samples we observed a lower percentage of degranulating NK cells in NK subsets expressing NKG2A (NKG2A+ matched; NKG2A+ mismatched; or NKG2A+ KIR−) as compared to their counterparts without NKG2A (NKG2A− matched; NKG2A− mismatched; or NKG2A− KIR−) and these were all in the group of NK cells expressing mismatched KIRs. These data demonstrated that the presence of NKG2A on NK cells did not seem to have an inhibitory effect on the response of IL-2-activated NK cells against HLA-class I competent cell lines expressing low levels of HLA-E. Moreover, for the subsets expressing no- or matched KIRs, the NKG2A positive cells performed even slightly better than their NKG2A negative counterparts.

**Figure 3:**
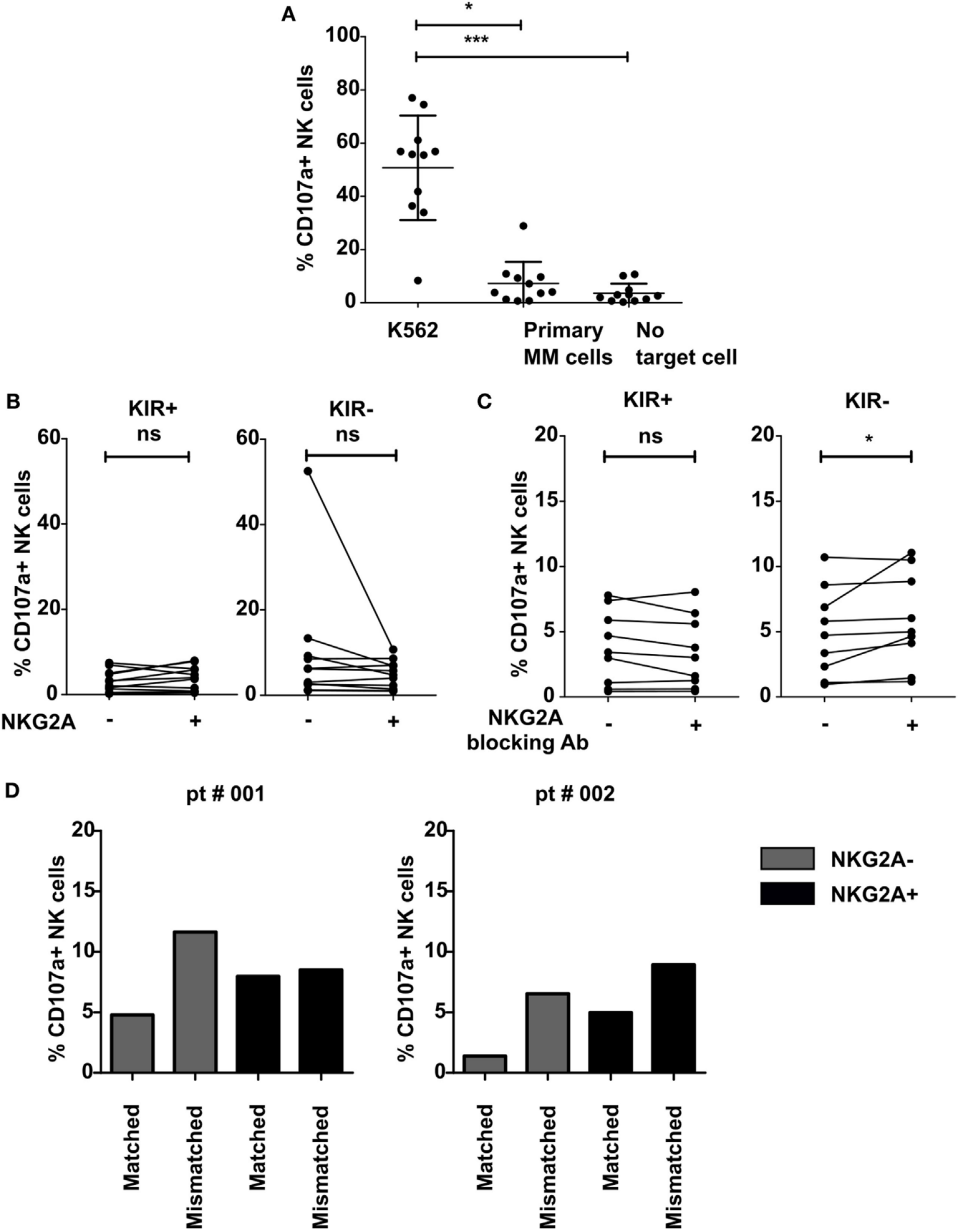
Effect of NKG2A expression on natural killer (NK) cells on NK cell activation against multiple myeloma (MM) cell lines and primary MM cells. IL-2-activated NK cells were co-cultured with K562 cell line **(A)**, primary MM **(A–D)**, or without target cells **(A)** for 4 h in a degranulation assay with or without an NKG2A blocking antibody **(C)**. Flow cytometric analysis was used to subtype NK cells based on their expression of NKG2A and killer immunoglobulin-like receptors (KIR). Degranulating NK cells were denoted as CD107a+ NK cells. Each dot represents an average of a technical replicate. **(A–C)** *n* = 10 independent experiments with samples from 10 different MM patients as target cells. **(D)** *n* = 2 different myeloma patients used as target cells (ns, not significant, *p < 0.05, **p < 0.01, ***p < 0.001).

To further investigate the functional relevance of NKG2A, we incubated IL-2-activated NK cells with primary MM cells known to express relatively high levels of both the classical HLA-class I and nonclassical HLA-class I (HLA-E) molecules (15). This revealed that the NK cells were highly activated by K562 cell line used as positive control, but not by the primary MM cells or in the absence of target cells (Figure 3A). For both KIR positive and KIR negative subsets, we did not observe any difference in NK cell degranulation between NKG2A expressing vs non-expressing NK cells both in the presence of primary MM cells (Figure 3B) or in the absence of primary MM cells (Figure S5 in Supplementary Material). As the level of degranulation in response to primary MM cells was very low and this could have blunted analysis of inhibitory effects by NKG2A, we blocked the HLA-E–NKG2A interaction with an NKG2A blocking antibody (Figure 3C) or with an anti HLA-E antibody (Figure S6 in Supplementary Material). To study the effect in more detail, we analyzed the effect of blocking on different subsets of NK cells. However, because the blocking NKG2A antibody has the same epitope with the fluorochrome-labeled NKG2A antibody, we could not visualize the NKG2A+ population and, therefore, we subtyped our NK cells into KIR+ and KIR− subtypes. Our results demonstrated that there was no effect of NKG2A blockade on the KIR+ subset and only a very small effect on the KIR− subset where we saw a small increase of CD107a+ NK cells in 2 out of 9 samples (*p* < 0.05) (Figure 3C). In this analysis, all KIRs were matched with the primary MM cells as the patients were C1+, C2+, and Bw4+. In two samples, there was a genetic discrepancy between KIRs and HLA on the primary MM cells enabling us to subgroup NK cells based on the KIR–ligand matched/mismatched status and to investigate whether KIR–ligand interaction played a bigger role than NKG2A–HLA-E interaction in inhibiting NK cells activity. Although the number of patients was not sufficient to perform statistical analysis, this suggested that the NKG2A− KIR–ligand mismatched cells degranulated more than the NKG2A− matched counterpart (Figure 3D). NKG2A+ KIR–ligand mismatched NK cells, however, were equally activated in one patient and more activated in one patient compared to the matched subset.

Altogether, the data from MM cell lines and primary MM cells demonstrated that NKG2A did not seem to play a major inhibitory role in the anti-MM response of high dose IL-2-activated NK cells.

### Daratumumab Triggers ADCC in All NK Cell Subsets Which Is Irrespective of the NKG2A Status

**Figure 4:**
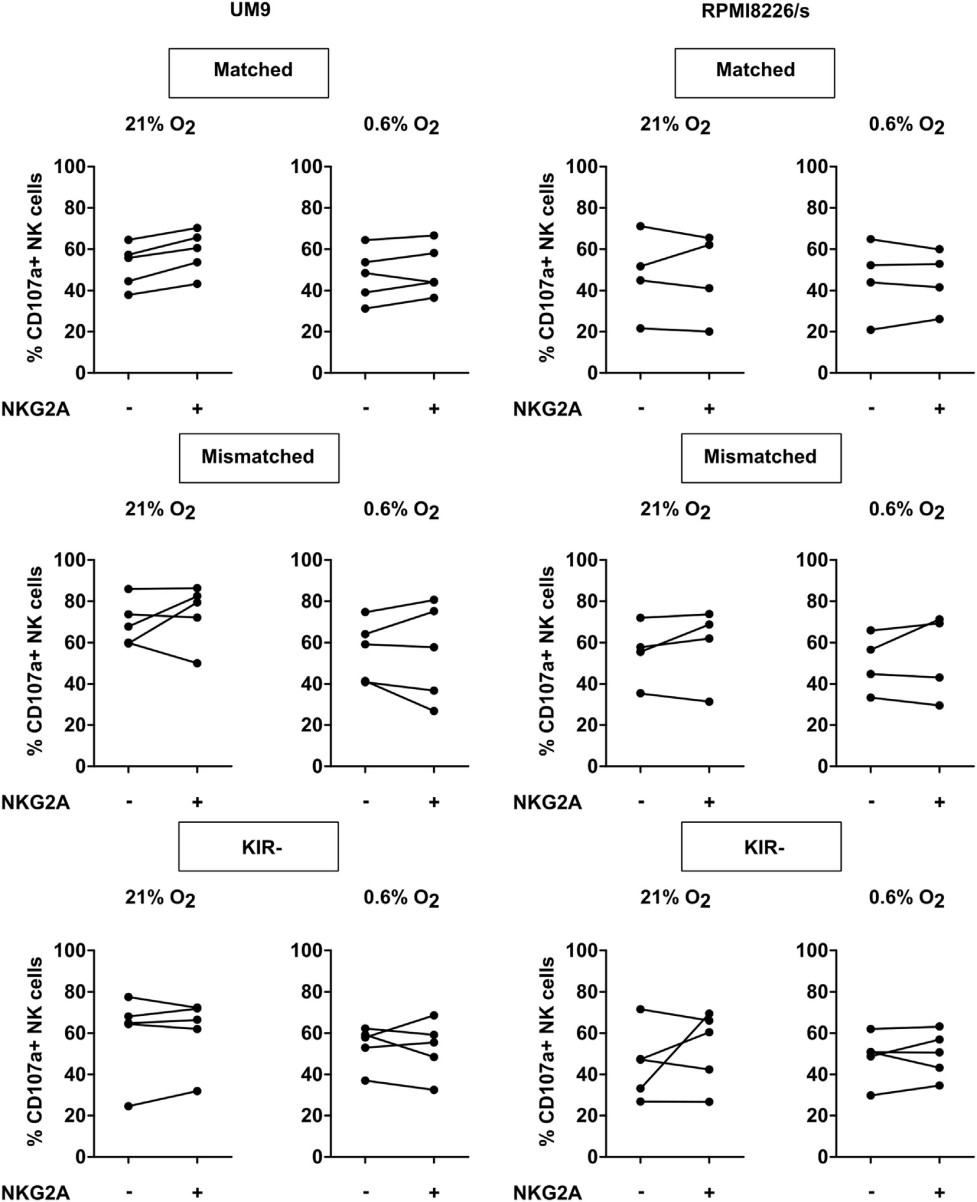
Effect of NKG2A co-expression on daratumumab-induced antibody-dependent cell-mediated cytotoxicity. UM9 or RPMI8226/s cells were pre-incubated with daratumumab for 30 min before adding IL-2-activated natural killer (NK) cells at a 1:1 E:T ratio. A degranulation assay was performed for 4 h at 21% O_2_ or 0.6% O_2_. Flow cytometric analysis was used to subtype NK cells based on their expression of NKG2A and killer immunoglobulin-like receptors (KIR). Degranulating NK cells were denoted as CD107a+ NK cells. Each dot represents an average of a technical replicate per donor. *n* = 5 independent experiments with five different donors and two different cell lines.

Myeloma-specific monoclonal antibodies that trigger NK cell-mediated ADCC are a potent way to boost the NK antitumor response, and also in this setting, we studied the role of NKG2A in controlling NK activation. To trigger ADCC, UM9, and RPMI8226/s, two MM cell lines that highly expressed CD38 were pre-incubated with daratumumab followed by a CD107a assay with IL-2-activated NK cells at 21 or 0.6% O_2_ and analysis of degranulation of individual NK cell subsets. This revealed that the addition of daratumumab clearly triggered NK cell degranulation for all subsets, at 21% O_2_ as well as at 0.6% O_2_ (Figure S7 in Supplementary Material). For UM9, the median fold enhancement ranged from 2.5- to 19-fold at 21% O_2_ and 2.0- to 44.5-fold at 0.6% O_2_. For RPMI8226/s, the median fold enhancement ranged from 1.9- to 11.4-fold at 21% O_2_ and 3.5- to 11.6-fold at 0.6% O_2_. Analysis of CD107a+ NK cells per subset subsequently demonstrated that there was no difference in degranulation between subsets expressing NKG2A vs subsets lacking NKG2A (Figure 4).

**Figure 5:**
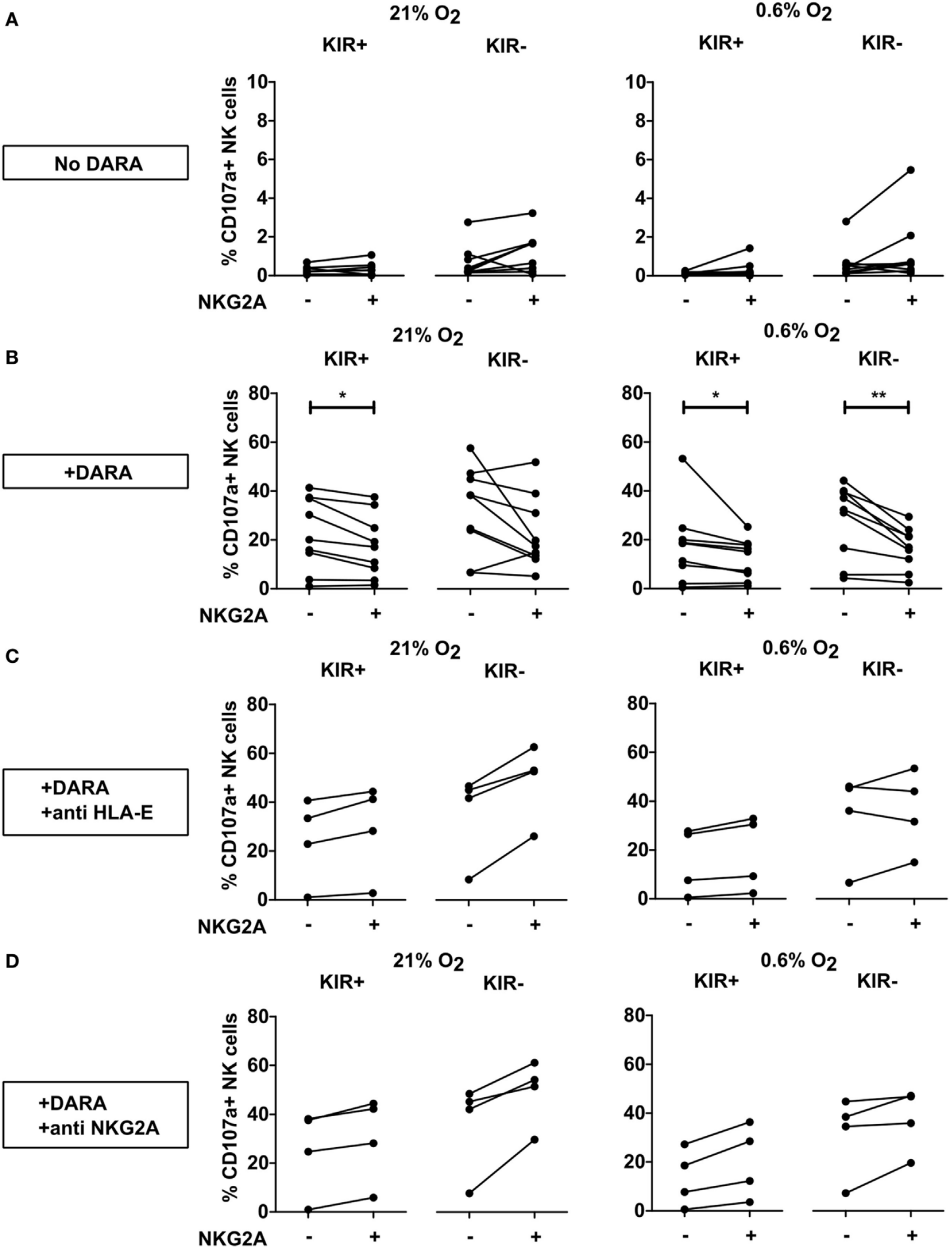
Effect of NKG2A and killer immunoglobulin-like receptor (KIR) expression on daratumumab (DARA)-induced natural killer (NK) cell fratricide. NK cells were incubated at 21% O_2_ or 0.6% O_2_ for 5 h in the absence **(A)** or presence of DARA **(B)**, or DARA and anti HLA-E **(C)** or DARA and anti-NKG2A **(D)**. Flow cytometry was used to subtype NK cells based on their expression of NKG2A and KIRs. Degranulating NK cells were denoted as CD107a+ NK cells. Each dot represents the average of a technical replicate (*p < 0.05, **p < 0.01). *n* = 9 different donors **(A,B)** or four donors **(C,D)**.

Natural killer cells also express CD38 on their surface and previous studies showed that NK cells could kill each other *via* ADCC triggered by NK cell-associated daratumumab. Therefore, we also compared the response of the NKG2A positive vs negative NK cells for the KIR+ and the KIR− subsets in the absence of tumor target cells. For this, IL-2-activated NK cells were incubated without (Figure 5A) or with daratumumab (Figures 5B–D) for 4 h followed by analysis of CD107a expression by NK cell subsets at 21% or 0.6% O_2_. Without daratumumab, we showed that spontaneous NK cell degranulation was very low for all subsets. For KIR+ NK cells, both at 21% and 0.6% O_2_, we observed a lower percentage of degranulating NK cells in subsets co-expressing NKG2A (Figure 5B). For KIR− subsets, we only saw this in the condition at 0.6% O_2_. To determine whether this was truly due to NKG2A, we blocked HLA-E–NKG2A interaction with an antibody blocking either HLA-E or NKG2A. For all donors and in both the KIR+ and KIR− NK cell subsets, the level of degranulation of NKG2A positive subsets was higher than that of NKG2A negative subsets after blocking, except in one donor under hypoxia in the presence of anti HLA-E, NKGA+, KIR− showed lower percentage of degranulating NK cells (Figures 5C,D). This illustrates that NKG2A could inhibit daratumumab-induced fratricide.

**Figure 6:**
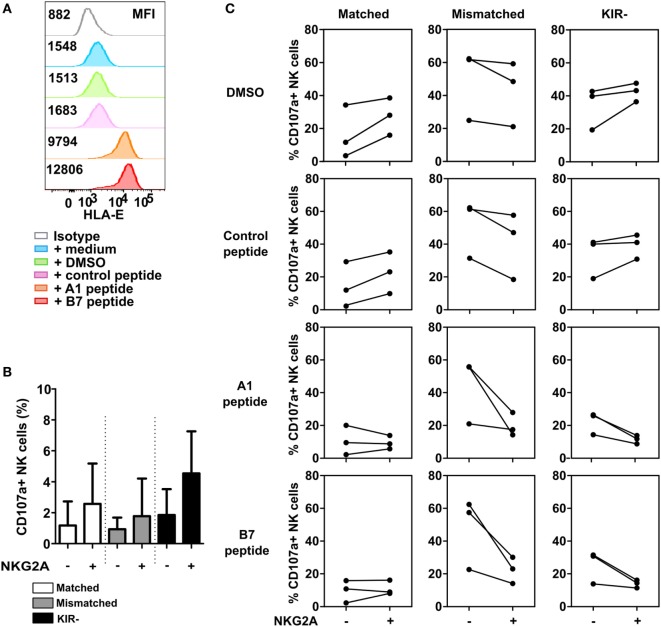
Inhibition *via* NKG2A is effective when a high level of HLA-E is present. **(A)** U266 cells were pre-incubated for 2 h with HLA-B7 peptide, HLA-A1 peptide, DMSO, control peptide (non-HLA-E binding), or medium. HLA-E expression of U266 is depicted in the histogram, with its corresponding median fluorescence intensity (MFI). **(B)** Spontaneous degranulation of IL-2 activated natural killer (NK) cells cultured for 13 h in the absence of target cells. **(C)** Degranulation of NK cells upon 13 h co-culture with peptide- or control-incubated U266 target cells. Degranulating NK cells were denoted as CD107a+ NK cells. Each dot in the graphs represents the average of a technical replicate for an individual donor. Error bars in **(B)** indicate SD. *n* = 3 different NK cell donors.

As highly activated NK cells express higher levels of HLA-E than the MM cell lines (Figure S3 in Supplementary Material), we hypothesized that the level of HLA-E might influence the potential of NKG2A to inhibit highly activated NK cells. To explore this, we performed a 4-h degranulation assay using IL-2-activated NK cells from three healthy donors against U266, a MM cell line expressing low levels of HLA-E. Prior to co-culture with NK cells, U266 cells were incubated with either medium, DMSO, control peptide, HLA-A1 peptide, or HLA-B7 leader peptide. The HLA-A1 or B7 peptides are derived from the leader sequence of HLA-class I and have been shown to bind HLA-E and enhance HLA-E surface expression (18). We observed that HLA-E was highly expressed on U266 cells upon peptide incubation, approximately sixfold (HLA-A1 peptide) and eightfold (B7 peptide) higher than the baseline expression (Figure 6A). In the absence of target cells (Figure 6B), NK cells subsets expressing NKG2A showed a higher degranulation compared to NK cell subsets not expressing NKG2A. For subsets expressing matched KIRs or no KIRs, NKG2A+ NK cells degranulated more than NKG2A− NK cells in the conditions where target cells were incubated without or with control peptide (Figure 6C). This was true for all three donors and in line with the data obtained with the other MM cell lines. For the KIR− subset, upregulation of HLA-E resulted in less degranulation in the NKG2A+ NK cells vs the NKG2A− cells, suggesting inhibition by NKG2A. For the matched KIR subset this effect was less pronounced. For the mismatch subset, we saw a lower percentage of degranulating NKG2A+ NK cells vs NKG2A− NK cells in all conditions. This supports the NK cell fratricide data (Figure 5) and together illustrates that NKG2A can inhibit high dose IL-2-activated NK cells but whether or not this occurs depends on the exact NK cell subset and presumably also on the type of target cells and the level of HLA-E on the target cells.

## Discussion

We envision that the ideal NK cell product for cancer treatment would be a large number of highly activated NK cells which can withstand the suppressive tumor microenvironment. Therefore, to refine NK cell-based immunotherapy, we focus our investigations on NK cell *ex vivo* expansion and strategies to enhance activation and to reduce inhibition of NK cells. Since the IL-2-activated NK cell *ex vivo* expansion protocols could result in a higher percentage of NKG2A expressing NK cells (19, 20), we performed, in the current study, an in-depth analysis of the influence of NKG2A expression on different NK cell subsets in response to MM cells showing that the inhibitory potential of NKG2A, for activated NK cells, depends on the exact subset of NK cells and the HLA-E context of the target cell.

NKG2A has a dual function in NK cells, on the one hand, it is required for NK cell licensing, but it also acts as an inhibitory receptor to control the activation threshold of the NK cell and to avoid autoimmunity (3). Here, we show that high dose IL-2-activated NK cells expressing NKG2A degranulated more vigorously than subsets not expressing NKG2A. We observed this irrespective of the presence of KIR and in response to HLA-deficient K562 leukemia cells and to a lesser extent against HLA competent MM cell lines. For K562, this is in line with previous studies in both mice (4, 5) and human (6). These studies showed that for unactivated NK cells, the more inhibitory receptors an NK cell expresses, the better the NK cell is licensed, and the more potently it can respond to HLA-class I deficient tumors. However, for HLA competent tumors, the presence of NKG2A could be a disadvantage due to the inhibitory signaling resulting from the NKG2A–HLA-E interaction. Although some tumor cells downregulate surface expression of HLA-class I molecules, tumors can also maintain or even enhance HLA-class I (21, 22). We and others previously demonstrated that MM cell lines and primary MM cells express HLA-class I and HLA-E on their surface (15, 23). Nevertheless, in the present study, NKG2A expression on high dose IL-2-activated NK cells did not result in a reduced activation. On the contrary, the presence of NKG2A seemed to be more advantageous for the NK cell response, especially against MM cell lines. Although NKG2A expressing vs non-expressing subsets might differ in more features than only NKG2A, our data suggest that for this high dose IL-2-activated NK cells the benefit of better licensing due to NKG2A seemed stronger than the inhibitory effects provided by this receptor.

Even in response to primary MM cells, despite relatively high HLA-E levels, no inhibitory effects of NKG2A were observed. As this could have been caused by the very low level of NK cell degranulation, we also blocked the NKG2A receptor using monoclonal antibodies recognized for their capacity to block HLA-E or NKG2A. The effect of blocking was very minor and only present in KIR+ subsets which was in contrast to a previous study where Monalizumab, a clinical grade NKG2A blocking antibody, improved the cytotoxicity of low dose (250 U/ml) activated KIR− NKG2A+ NK cells against a variety of primary tumor cells (10). One of the differences with our study was that Ruggeri et al. used NKG2A+ KIR− NK cell clones, while we used a heterogeneous population of NK cells. The percentages of NKG2A+ KIR− NK cells among our donors varied between 11.6 and 51.8% of total NK cell populations (Table S1 in Supplementary Material) which might have influenced the blocking capacity of the antibody. In our previous study (15), also using whole NK cells but non-activated, we showed that there was an increased percentage of overall CD107a+ NK cells when we blocked HLA-E/NKG2A interaction using an HLA-E antibody. Therefore, another reason could be the difference in activation and or licensing status of the NK cells as we used in the current study healthy donor-derived NK cells pre-activated with a high dose of IL-2 (1,000 U/ml), while Ruggeri et al. used only 250 U/ml for activation. This suggests that the activation status of the NK cells is important for whether or not NKG2A can mediate strong inhibitory effects on NK cells. Importantly, this also suggests that if NK cells are sufficiently activated, e.g., *via* cytokine activation, the co-expression of NKG2A is not *per se* detrimental.

The potency of NKG2A to inhibit NK cells can also be influenced by the HLA-E expression level on the target cell. Previously, inhibition *via* KIR has been shown to have a linear relation with HLA-class I, meaning that the more of the ligand is expressed the more inhibition is mediated *via* the receptor (24). For NKG2A this seems different as the same group also showed that inhibition by NKG2A occurs only when HLA-E levels are above a certain threshold and the strength of the inhibitory signal could not be further amplified by increasing expression levels of the HLA-E ligand (24). Although the number of individuals was limited, in our experiments, peptide-induced HLA-E expression made NKG2A+ KIR− less responsive than their NKG2A− KIR+ counterparts. In line with this, blockade of the NKG2A co-receptor CD94 has been shown to enhance the response of highly activated NKG2A+ NK cells against a cell line transgenically expressing very high levels of HLA-E but not against primary ALL cells expressing an intermediate level of HLA-E (25). In addition, we observed that NKG2A positive NK cells, expressing high levels of HLA-E, mediated less daratumumab-induced fratricide than NKG2A negative NK cells which could be reversed by adding anti-HLA-E or anti-NKG2A. Highly activated T cells express increased levels of HLA-E and this protects them from killing by NKG2A positive NK cells (26). We now show that this is also true for highly activated NK cells. Furthermore, we show that NKG2A has the potential to inhibit highly activated NK cells but that this depends on the exact setting and that activated NK cells may have a different threshold for HLA-E than unactivated NK cells.

Another important point to take into account in the design of NK cell immunotherapy is that NK cells will have to function in a suppressive tumor microenvironment. TMEF, such as hypoxia, lactate, prostaglandin E2, and others have been shown to dampen NK cell anti-tumor responses through several mechanisms summarized in Ref. (27). We, therefore, evaluated the role of NKG2A also in the presence of factors from the TME but did not see very obvious differences with the data obtained under normal control conditions. In addition, we did not observe changes in NKG2A expression on the NK cells, possibly because the 4 h incubation was relatively short to induce changes on IL-2-activated NK cells by hypoxia. We realize that our *in vitro* set up does not fully reflect the complexity of the *in vivo* TME, and several other studies in human or mice did show that the TMEF could lead to NK cell phenotypic change (28–30). Furthermore, we demonstrated in an earlier study that HLA-E levels on MM tumor cells are increased upon *in vivo* growth in the BM of immunodeficient mice as compared to *in vitro* passaged cells (15). Moreover, a recent paper has shown that under hypoxia and glucose deprivation, HLA-E can be upregulated in both human and mouse tumor cells as a result of microenvironmental stress (31). It would, therefore, be valuable to determine the effect of NKG2A on high dose IL-2-activated NK cells in an *in vivo* MM model or after longer exposure of hypoxia and or other TMEF.

Importantly, our data show that, for high dose IL-2-activated NK cells, all subsets can get activated by tumor cells in the context of laboratory setting mimicking tumor microenvironment. This is also true for the presumed “hyporesponsive” subset not expressing any licensing inhibitory receptor. Moreover, the addition of daratumumab, to trigger ADCC, even improved the response of this hyporesponsive subset to a level comparable to that of subsets expressing NKG2A or KIR. This is important as it illustrates that with potent activation all subsets could contribute to tumor clearance. Nevertheless, under all conditions, subsets expressing KIRs that were mismatched with the HLA ligands on the target cell had the highest level of activation, both in response to the MM cell lines as well as in response to primary MM. This emphasizes the relevance to select KIR–ligand mismatched NK cell donors or to use a KIR blocking antibody like lirilumumab. For NKG2A, selection of mismatched donors is not a feasible strategy, but, the interaction between NKG2A and HLA-E can be blocked with a clinically available monoclonal antibody (monalizumab). However, in case of an expanded NK cell product, where NK cells received a cocktail of cytokines, such as IL-2 or IL-15 providing strong activation signals, or in the situation where an ADCC-triggering antibody is used, this may not be very useful as these NK cells may be not severely inhibited by NKG2A. Additionally, blockade could even be detrimental. Therefore, a better understanding of the conditions leading to HLA-E expression in relation to the inhibitory effects *via* NKG2A, would be useful to predict for which patients blockade of NKG2A with monalizumab would be beneficial.

## Ethics Statement

Blood from healthy volunteers was obtained under a general agreement with the university hospital for blood withdrawal from healthy volunteers used for reference values and not specified per study. All participants gave written informed consent. Primary MM cells were obtained from the department of cytogenetics as a leftover material from a patient subject to a cytogenetic examination. Under the Dutch law on Research Involving Human Subject, leftover materials from a patient can be used for research and are waived from individual patient’s consent. The use of buffy coats, being a by-product of a required Medical Ethical Review Committee (METC) procedure, does not need ethical approval in the Netherlands under the Dutch Code for Proper Secondary Use of Human Tissue.

## Author Contributions

All authors listed have made a substantial, direct, and intellectual contribution to the work and approved it for publication.

## Conflict of Interest Statement

GB is Chief Executive Officer/Chief Medical Officer/Co-founder of CiMaas, BV, Maastricht, The Netherlands. CiMaas is producing an *ex vivo* expanded NK cell product that will be used to treat myeloma patients. The other authors declare no conflict of interest.
