# Supplementary material for: NKG2A Expression Is Not per se Detrimental for the Anti-Multiple Myeloma Activity of Activated Natural Killer Cells in an In Vitro System Mimicking the Tumor Microenvironment
Source: Front Immunol. 2018 Jun 22;9:1415. doi: 10.3389/fimmu.2018.01415 (PMC6023990; doi:10.3389/fimmu.2018.01415)
Supplement: Supplementary file 1 [file presentation_1.PDF]

Supplementary Material

NKG2A Expression is Not per se Detrimental for the Anti-Multiple Myeloma Activity of Activated Natural Killer Cells in an In Vitro System Mimicking the Tumor Microenvironment

Niken M. Mahaweni[1,2], Femke A. I. Ehlers[1,2], Subhashis Sarkar[1], Johanna W. H. Janssen[3], Marcel G. Tilanus[2], Gerard M.J. Bos[1], Lotte Wieten[2*]

*** Correspondence:** Dr. Lotte Wieten: l.wieten@mumc.nl

1. Supplementary Figures and Tables

1.1 Supplementary Figures

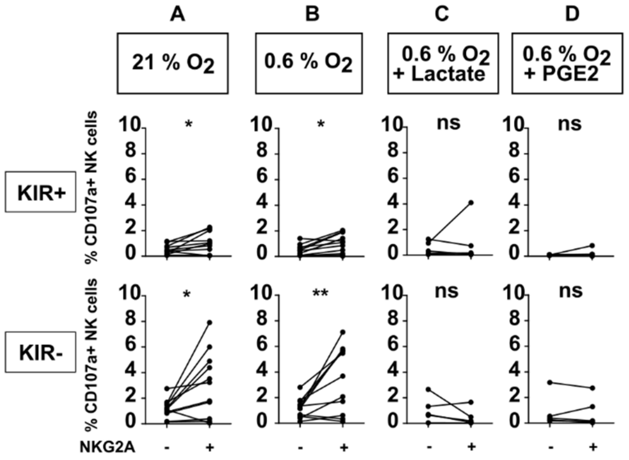

Sup Fig 1. Spontaneous NK cell degranulation in the presence of different microenvironment factors. NK cells were cultured without target cells for 4 hours at 21 (A) or 0.6 % O_2 (B), or combinations of 0.6 % O_2 and 50 uM lactate (C), or 100 ng/mL PGE2 (D). Flow cytometry was used to subtype NK cells based on their expression of NKG2A and KIRs. The percentage of degranulating NK cells is shown as % CD107a+ NK cells. Each dot represents an average of a technical replicate from an individual NK cell donor. (A) and (B) n = 11 donors, (C) and (D) n = 5 donors tested in independent experiments (* = p<0.05, ** = p<0.01)

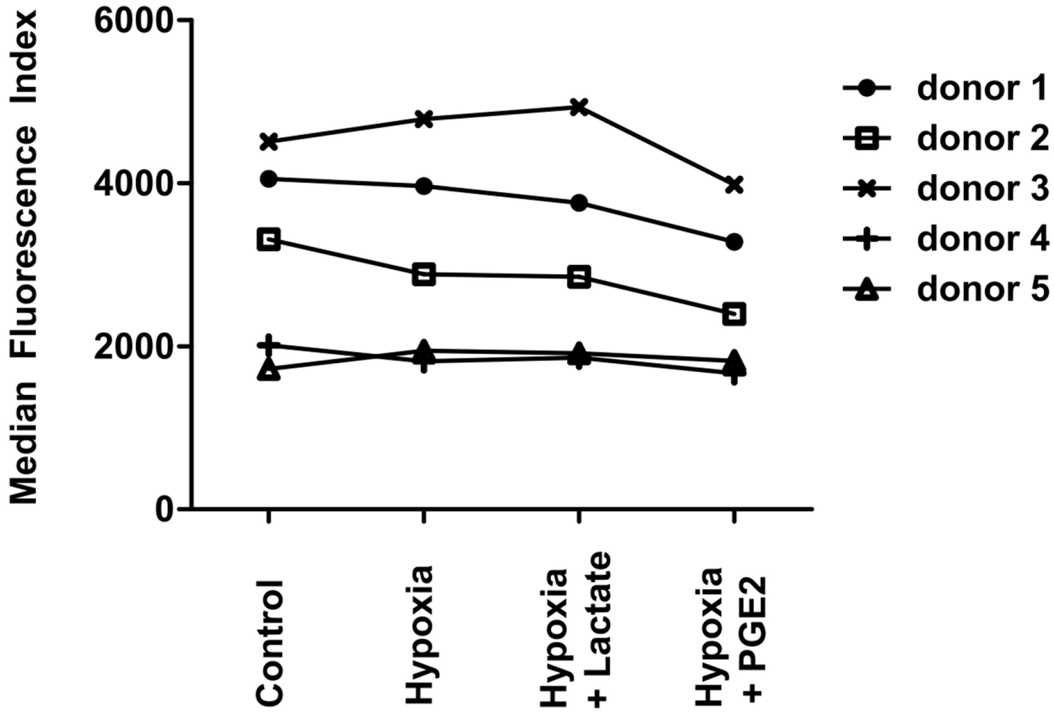

Sup Fig 2. NKG2A expression on NK cell is not affected by hypoxia, lactate, PGE2, or the combinations. NK cells were cultured for 4 hours in the presence of 21% or 0.6% O_2, or the combination of 0.6% O_2, and 50 mM lactate or 100 ng/mL PGE2. Each dot represents an average of a technical replicate forthe different conditions. n = 5 independent experiments

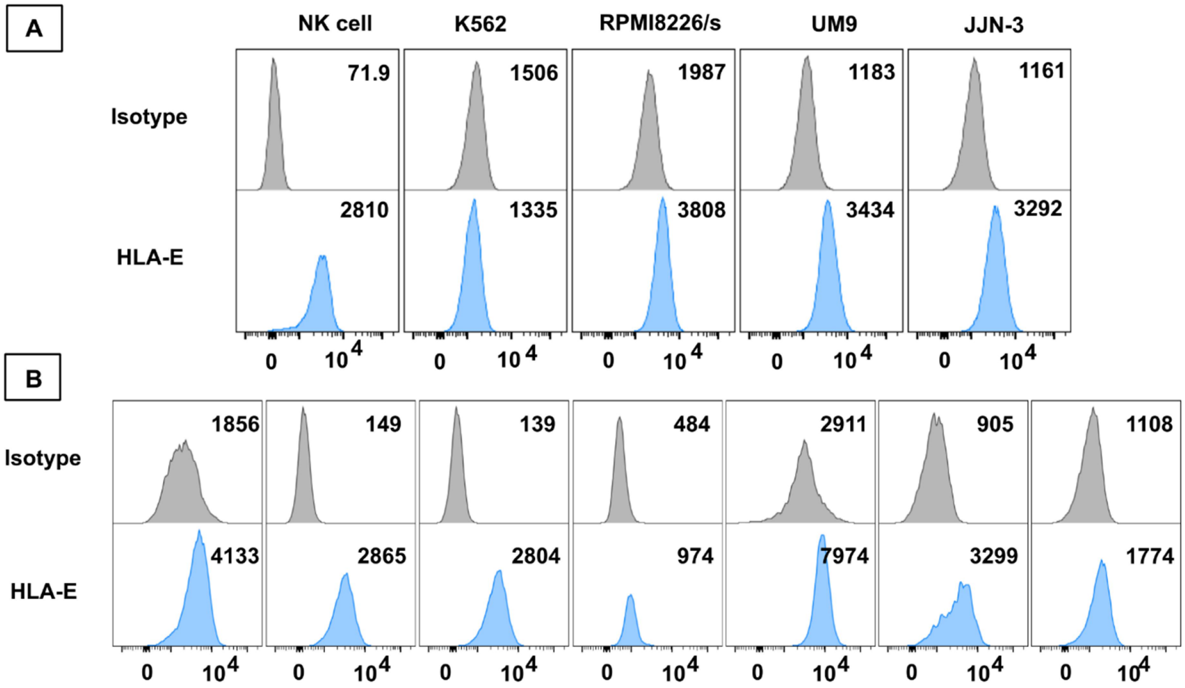

Sup Fig 3. HLA-E expression on different cell types. A) IL-2 activated NK cells, K562 or MM cell lines (UM9, RPMI8226/s or JJN-3 cells) or B) primary MM cells were stained with HLA-E antibody or isotypes as control or unstained. Each plot of primary MM cells represent 1 MM patient. Flow cytometric analysis was used to determine the expression of HLA-E. A median fluorescence intensity (MFI) is displayed in each plot.

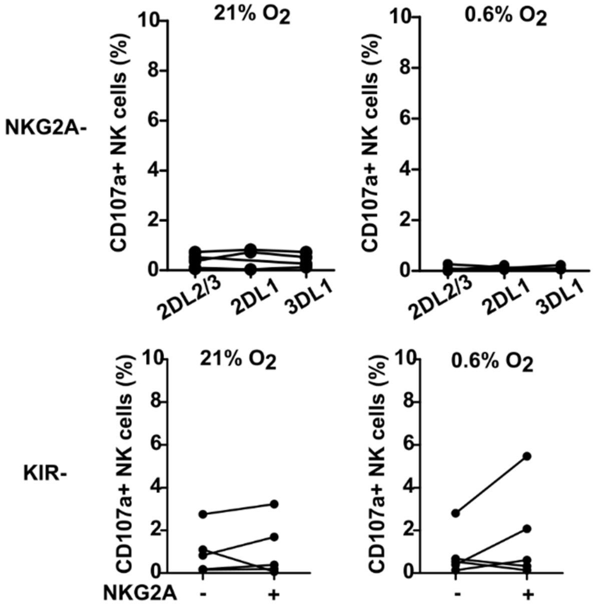

Sup Fig 4. Spontaneous NK cell degranulation per subset. NK cells were cultured without target cells (MM cell lines) for 4 hours in the presence of 21% or 0.6% O_2. The percentage of degranulating NK cells was denoted as CD107a+ NK cells. The subsets of 2DL2/3, 2DL1, and 3DL1 were gated based on NK cells expressing only one of the KIR receptors and lacking NKG2A expression. The subset of NKG2A+ KIR- was gated based on NK cells expressing only NKG2A receptor and missing all other KIR receptors. The subset of NKG2A- KIR- was gated based on NK cells expressing neither of NKG2A nor KIR receptors. n = 5 independent experiments

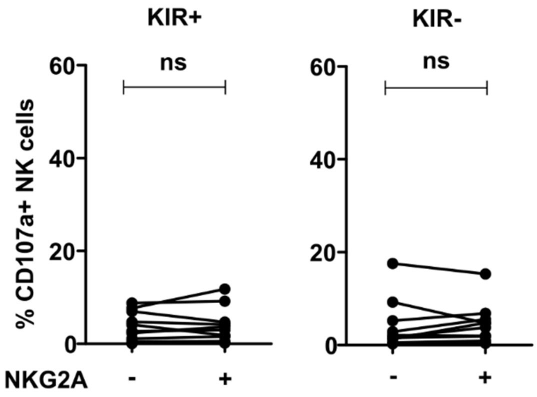

Sup Fig 5. Spontaneous NK cell degranulation based on NKG2A expression. NK cells were cultured without target cells (primary MM cells from patients) for 4 hours in the presence of 21% O_2. The percentage of degranulating NK cells was denoted as CD107a+ NK cells. The subset of NKG2A+ KIR- was gated based on NK cells expressing only NKG2A receptor and missing all other KIR receptors. The subset of NKG2A- KIR- was gated based on NK cells expressing neither of NKG2A nor KIR receptors.

n = 10 independent experiments

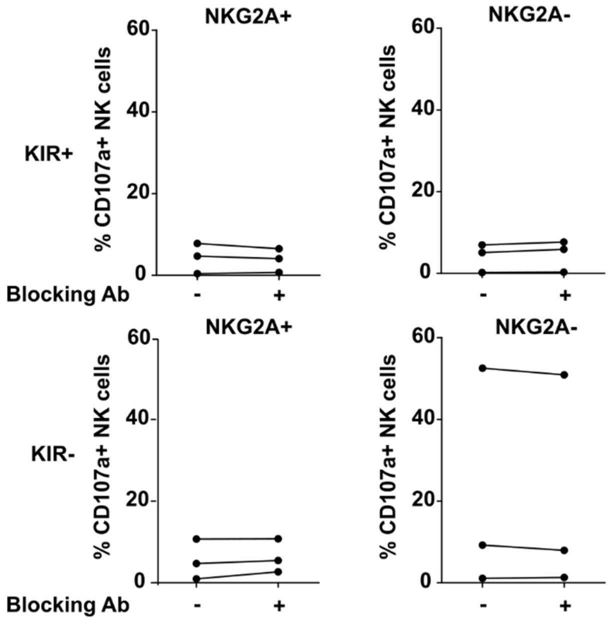

Sup Fig 6. Effect of HLA-E blocking on NK cell degranulation against primary MM cells. IL-2 activated NK cells were co-cultured with primary MM cells in a 1:1 E;T ratio in the presence of 10 µg/mL anti-HLA-E antibody for 4 hours in the presence of 21% O_2. Flow cytometric analysis was used to determine the NK cell degranulation (denoted by %CD107a+ NK cell). n = 3 independent experiments with three different MM patients.

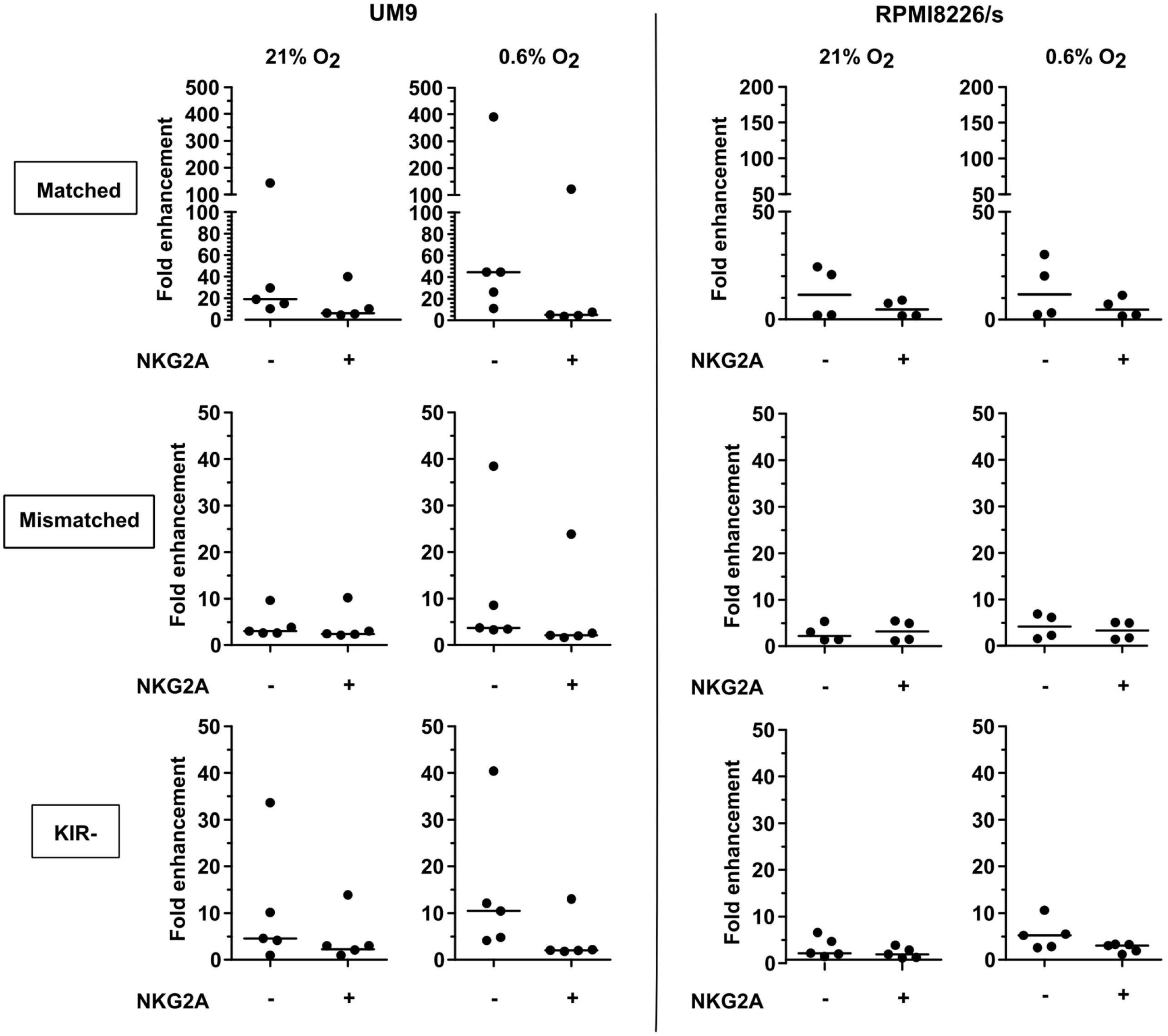

Sup Fig 7. Daratumumab enhanced degranulation of all NK subsets both at 21% or 0.6 % O_2. UM9 or RPMI8226/s cells were pre-incubated with daratumumab or medium for 30 minutes before adding IL-2 activated NK cells in a 1:1 E:T ratio. A degranulation assay was performed for 4 hours in the presence of 21 or 0.6% O_2. Flow cytometry was used to subtype NK cells based on their expression of NKG2A and KIRs. The fold increase in the percentage of CD107a+ NK cells in the presence of daratumumab for each subset was calculated by dividing the percentage of CD107a+ NK cells in the presence of daratumumab by the percentage of CD107a+ NK cells in the absence of daratumumab. Vertical bars shown in the plots are the median.

n = 5 experiments with 5 donors

1.2 Supplementary Table

Donor	**NKG2A+ (%)**		**NKG2A- (%)**	
	Total NKG2A+	NKG2A+ KIR-	Total NKG2A-	NKG2A- KIR-
A	42.9	30.3	54.4	14.5
B	29.4	16.1	68.0	15.4
C	81.4	51.8	16.7	5.3
D	27.7	18.4	62.4	26.2
E	54.3	42.5	37.9	18.1
F	42.8	31.7	49.8	35.2
G	54.7	18.9	39.1	4.5
H	52.1	34.3	33.6	13.4
I	66.2	36.6	29.3	3.1
J	19.2	11.6	77.8	14.0

Sup Table 1. The proportion of NKG2A+ KIR- cells and NKG2A- KIR- cells from different donors. NK cells were stained with fluorochrome-labeled antibodies targeting NKG2A, KIR2DL2/3, KIR2DL1, and KIR3DL1 receptor. The expression of receptors was measured by flow cytometry. The percentages presented in the table were calculated from total NK cells (CD3-CD56+).

n = 10 donors
